# Assessment of Biomechanical Response to Fatigue through Wearable Sensors in Semi-Professional Football Referees

**DOI:** 10.3390/s21010066

**Published:** 2020-12-24

**Authors:** Luigi Truppa, Michelangelo Guaitolini, Pietro Garofalo, Carlo Castagna, Andrea Mannini

**Affiliations:** 1 The BioRobotics Institute, Scuola Superiore Sant’Anna, 56127 Pisa, Italy; michelangelo.guaitolini@santannapisa.it (M.G.); a.mannini@santannapisa.it (A.M.); 2 Department of Excellence in Robotics & AI, Scuola Superiore Sant’Anna, 56127 Pisa, Italy; 3 TuringSense EU Lab s.r.l., 47121 Forlì, Italy; pietro.garofalo@turingsense.com; 4 School of Sport and Exercise Sciences, Università di Tor Vergata, 00118 Rome, Italy; castagnac@libero.it; 5 Italian Football Federation (FIGC) Technical Department, Football Training and Biomechanics Laboratory, 50135 Firenze, Italy; 6 IRCCS Fondazione don Carlo Gnocchi, 50143 Firenze, Italy

**Keywords:** fatigue detection, counter-movement jump, wearable inertial sensors, football, biomechanics

## Abstract

Quantifying muscle fatigue is a key aspect of everyday sport practice. A reliable and objective solution that can fulfil this task would be deeply important for two main reasons: (i) it would grant an objective indicator to adjust the daily training load for each player and (ii) it would provide an innovative tool to reduce the risk of fatigue-related injuries. Available solutions for objectively quantifying the fatigue level of fatigue can be invasive for the athlete; they could alter the performance or they are not compatible with daily practice on the playground. Building on previous findings that identified fatigue-related parameters in the kinematic of the counter-movement jump (CMJ), this study evaluates the physical response to a fatigue protocol (i.e., Yo-Yo Intermittent Recovery Test Level 1) in 16 football referees, by monitoring CMJ performance with wearable magneto-inertial measurement units (MIMU). Nineteen kinematic parameters were selected as suitable indicators for fatigue detection. The analysis of their variations allowed us to distinguish two opposites but coherent responses to the fatigue protocol. Indeed, eight out of sixteen athletes showed reduced performance (e.g., an effective fatigue condition), while the other eight athletes experienced an improvement of the execution likely due to the so-called Post-Activation Potentiation. In both cases, the above parameters were significantly influenced by the fatigue protocol (*p* < 0.05), confirming their validity for fatigue monitoring. Interesting correlations between several kinematic parameters and muscular mass were highlighted in the fatigued group. Finally, a “fatigue approximation index” was proposed and validated as fatigue quantifier.

## 1. Introduction

Muscle response after a preload stimulus is the result of its adaptability. Indeed, muscles are characterized by an intrinsic plasticity that allows them to conveniently calibrate their force, endurance and contractile speed according to the effort they are subjected to. This phenomenon is particularly evident in sport, where the specific adaptation of the muscle tissue after consecutive training sessions allows the athlete’s performance to be maximized [1] (i.e., improved jumps [2]). In addition to a long-term plasticity, the muscles are able to perform a temporary adaptation during and after endurance exercises and activities involving high speed and power (i.e., a football match) by recruiting an increased number of higher order motor units [3]. This process is called Post-Activation Potentiation (PAP), and it generally causes an increase in the low-frequency force exerted by the athlete during an endurance exercise, but it can also increase the force at higher frequencies in concentric versus isometric contractions (e.g., jumping, swimming and cycling) [4]. On the other hand, since muscles cannot rely on boundless sources of energy, they undergo a process of fatigue, which goes against the positive effects of PAP. Indeed, fatigue is defined as the inability to keep the athletic performance above a certain level as the result of an excessive workload due a gradual loss of muscular force [5,6]. Therefore, total muscular response during an endurance exercise is determined by the balance between these two opposite but simultaneous phenomena [3]. Even though scientific literature assumes that the above balance is affected by training experience, rest period length and intensity of the conditioning exercise performed, the topic remains still open and ambiguous [3].

While Post-Activation Potentiation produces temporary benefits in the athlete’s performance, fatigue could lead to severe muscle damages. According to Ekstrand et al., fatigue-related injuries constitute 31% of all injuries, causing more than 27% of total injury absence in the club considered in their study [7]. Since muscle injuries tend to occur mostly at the end of the two halves of a football match ([7,8]) and they are more frequent during the matches than in training sessions [7], fatigue could be reasonably included among the first causes of an increased risk of injury. Moreover, susceptibility to muscle strain injury is greater during explosive ballistic actions (i.e., acceleration and deceleration during sprinting) [8]. In this context, the prior recognition of fatigue could play a key role in injuries prevention.

Several methods were used to quantify training load, such as questionnaires, diaries, physiological monitoring and direct observation [9]. Questionnaires and diaries allow data to be obtained by directly interviewing athletes (i.e., the session Rate of Perceived Exertion [10]). They are easy to manage, cost effective and compatible with training, but they are extremely subjective (as well as direct observations), thus limiting their effectiveness. More objective methods could be physiological measures (i.e., heart rate, oxygen consumption, lactate concentration, electromyography (EMG) and critical power), but they strongly depend on environmental conditions, diurnal changes, hydration status, altitude, age and medications [9]. In addition, even though measurement equipment is becoming increasingly miniaturized, the physiological monitoring still remains not very practical to carry out during training session. Moreover, surface EMG, which can provide real time measurements, fails to give certain information about the nature of muscle adjustments due to fatigue in vertical jump [11]. Another technique used in fatigue monitoring is muscle biopsy, which is highly invasive, painful, time consuming and expensive [12]. Alternatively, movement analysis based on various technologies (i.e., video-based analysis systems, semiautomatic multiple camera systems and global positioning systems) could be used [13], but it turns out to be time consuming and unfitting for daily fatigue monitoring.

Previous works suggested the possibility to identify fatigue markers in a non-invasive way by studying the athlete’s performance in counter-movement jump (CMJ), which is a well-established practice in sports providing information on both the fatigue in the athlete and his supercompensation due to a periodic training path [5,14,15,16]. Indeed, several studies on professional athletes have shown that fatigue condition influences both kinematics and performing strategy of the CMJ, inducing compensatory movements [17]. Starting from this, this work focused on the kinematic characterization of the eccentric phase of the jump, in which muscles exert a greater force, and the alteration could be more significant [18].

Specifically, the aim of this work is to study variations in human kinematic parameters after a football-specific fatigue protocol (e.g., Yo-Yo Intermittent Recovery Test Level 1—YYIR1) using wearable sensors and to analyse these variations so as to detect and quantify the fatigue condition in athletes. In other terms, the main hypothesis of the present study is that fatigue could alter kinematic parameters in semi-professional athletes during the execution of a simple gesture, such as the Counter-Movement Jump, and that wearables can be used to track their variation. In order to achieve this objective, a body sensor network based on a magneto-inertial measurement unit (MIMU) was used. MIMUs and wearables in general, in our view, are the key enabling technologies for an in-field fatigue assessment, which is extremely important in the daily practice of athletes and coaches. MIMUs are very versatile, cheap and small in size sensors. They can be used in uncontrolled environments too (enabling the possibility to test methods directly on the playground), but they are characterized by a minor accuracy compared to optoelectronic methods. For this reason, they require particular care with regard to computational strategies to extract reliable information from them. Nevertheless, previous studies validated MIMUs for jump kinematic analysis, proving that there is no statistical differences between the accuracy of these units and that of an optoelectronic system in parameters extracted during this specific gesture [19].

## 2. Materials and Methods

### 2.1. Subjects

Sixteen semi-professional male football referees (Sezione Associazione Italiana Arbitri di Firenze, Firenze, Italy. Age: 22.9 ± 3.9 years, height: 179.9 ± 7.6 cm, weight: 75.3 ± 8.7 kg, years of previous training: 12.9 ± 4.9 years) were involved in testing. Athletes, in accordance with the Helsinki protocol, signed an informed consent form and filled out a questionnaire to take part in the tests. No subject had a record of cardiovascular/respiratory disease or evidence of neuromuscular diseases in the twelve months prior this study. This study was approved by the Joint Ethics Committee of the Scuola Normale Superiore and Scuola Superiore Sant’Anna (Approval no. 4/2020).

### 2.2. Experimental Procedures

The acquisition protocol was conducted in a controlled environment (e.g., Italian Football Federation gymnasium in Coverciano, FI, Italy), and it consisted of the following steps:

- A 5 min warm-up phase of jogging on treadmill at fixed speed (i.e., 10 km/h);
- Pre-fatigue acquisition, in which MIMUs were placed on the participant’s body using elastic bands, and the athlete performed one series of 5 CMJs separated by a 10 s of rest time (at the end of this acquisition sensors were removed to facilitate the following phase);
- Fatigue phase induced in athletes by using the YYIR1 (which consisted of 2 × 20 m shuttle runs at increasing speeds, with a 10 s period of active recovery, allowing us to quantify the individual’s capacity to perform repeated intense exercise and to examine changes in performance [20]);
- Post-fatigue acquisitions in which the same CMJ test conducted in the pre-fatigue acquisition (i.e., 5 CMJs separated by a 10 s of rest time) was repeated after 5 min from the end of fatigue exercises.

**Figure 1:**
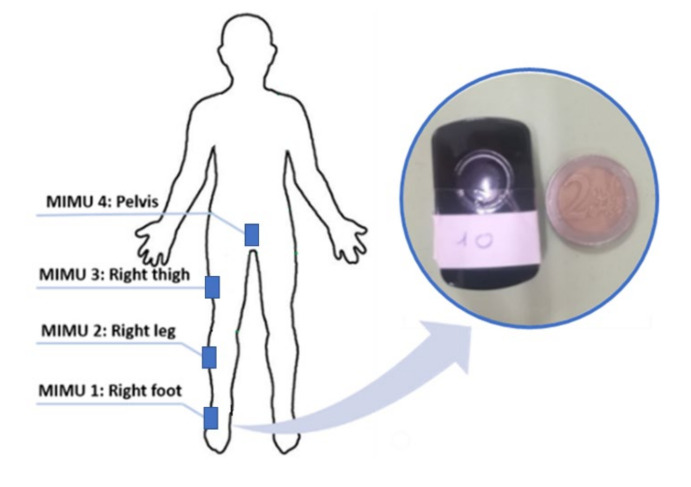
Magneto-inertial measurement units (MIMUs) placement sites.

Pre- and post-fatigue parameters were estimated from the most similar four jumps in terms of body centre of mass acceleration traces (by signals visual inspection) and then averaged of the relative series. The pre-fatigue acquisitions were used as a baseline to compare data from post-fatigue measurements. Data were acquired using a body sensor network of four MIMUs (TuringSense, Santa Clara, CA, USA. Acquisition frequency: 100 Hz) laterally placed on the right foot, right thigh, right leg and pelvis (Figure 1). The foot sensor was placed directly on the participants’ shoe. The right thigh and right leg MIMUs were placed in the middle point of the corresponding body segment directly on the skin. In order to limit the soft-skin and muscle contraction artifacts, these three sensors were placed laterally with elastic bands solidly tied. Finally, the pelvis unit was placed in correspondence to the fifth lumbar vertebrae using again an elastic band. MIMUs on the right lower limb were aligned with the corresponding body segment by exploiting a preliminary flex-extension movement [21] (i.e., functional calibration). This flex-extension movement allowed us to define the horizontal axis of the sensor by measuring the mean outputs of the gyroscopes and, then, to align it with the flex-extension axis of the corresponding body segment. As concerns the vertical axis of the sensors, it was estimated as the gravity vector measured during an N-pose and, then, aligned with the longitudinal axis of the corresponding body segment. Since most of CMJ outputs refer to the human body centre of mass (COM), the pelvis sensor was placed in correspondence to the fifth lumbar vertebra (L5), which represents a reasonable approximation of the human barycentre [14,22]. In addition to the kinematic parameters, physiological and anthropometric parameters were also measured (i.e., height, weight, blood lactate after fatigue induction) together with the distance covered during the YYIR1. Moreover, muscular mass was evaluated by exploiting James’ formula [23]. Given the existing variability among participants, the muscular mass was calculated to investigate its possible correlations with the proposed set of kinematic parameters. The blood lactate level was measured using the Accutrend Plus interferometer (Roche, Basilea, Switzerland).

### 2.3. Segmentation of the Jump

In order to estimate the correct acceleration of the body centre of mass, a preliminary alignment of the sensor with the gravity vector was performed by using the quaternions provided by the inertial unit located on the pelvis. After this initial alignment, gravity was subtracted from the acceleration of the body centre of mass to obtain its inertial acceleration. All the above parameters were estimated starting from this inertial acceleration and neglecting the influence of the gravity. This procedure allowed us to isolate only the contribution of the countermovement to the COM kinematic parameters. 

**Figure 2:**
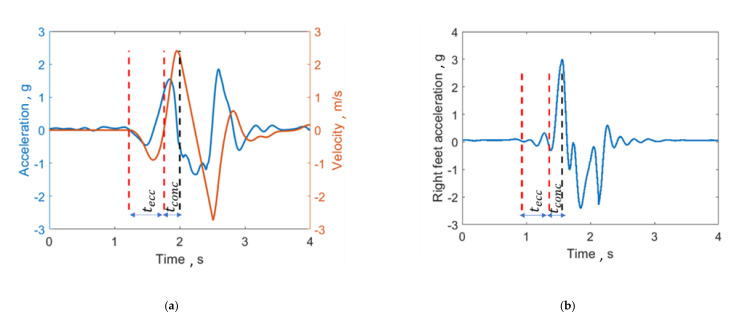
(**a**) Segmentation of the jump from acceleration and velocity of the body centre of mass. $t_{ecc}$ and $t_{conc}$ are the eccentric and concentric phases duration, respectively. The red dotted lines limit the eccentric phase of the jump, while the black one delimits the concentric phase. (**b**) Acceleration traces of the right foot, which is characterized by a peak in correspondence to the take off. The two negative peaks in the acceleration trace correspond to an extension movement of the foot immediately after the take off and before the landing, respectively. In particular, the first movement is the consequence of the boost phase that precedes the take-off, while the second one is an anticipatory movement carried out by the athlete for better landing on the ground.

The typical trace of the body centre of mass inertial acceleration in the vertical direction is shown in Figure 2: when the subject is still, his inertial acceleration is zero, then, when he performs the counter-movement, the inertial acceleration starts to decrease (it is negative). The eccentric phase of the jumps ends when the velocity of the centre of mass is zero (the subject is loading the following boost phase), while the concentric phase of the jump starts immediately after the eccentric one and ends when the feet rise from the ground. To understand when the feet detach from the ground, the MIMU on the right foot is used. Indeed, its acceleration is characterized by a peak when the foot takes off. Therefore, the jump was segmented as follows on “Matlab” (Mathworks, Natick, MA, USA)

- The start of the eccentric phase was identified by analysing the acceleration curve and automatically finding when it goes below a specific threshold (i.e., mean +3 standard deviations of the first 100 samples);
- The end of the eccentric phase corresponds to the zero crossing of the velocity (second red line in Figure 2);
- The end of the concentric jump coincides with the peak in the acceleration curve of the foot sensor. The peak of the curve was automatically detected by finding the maximum value of the acceleration.

### 2.4. Measurements

Fifteen of the nineteen considered parameters were classified as COM kinematic, while the remaining four as articular joint kinematic. The differentiation between the two kinds of parameters was carried out by considering the equations used for determining the above parameters: those coming from the integration/differentiation of the pelvis acceleration (Table 1) were classified as “COM kinematic” because they do not refer directly to the lower-limb joints analysis, while those obtained from the sensors quaternions (Equation (1)) were classified as “articular joint kinematic” because they refer directly to joint analysis (i.e., joints angles and angular velocities).

In turn, the COM kinematic parameters could be divided into four main groups:

- *Displacement parameters:* the mean displacement of the centre of mass during the eccentric phase (i.e., jump depth) is analysed in this work. In our opinion, monitoring the mean position of the barycentre could be useful to understand how much the athlete is loading the jump due to fatigue.
- *Power and energy parameters*: allow us to quantify the lower-limb explosive ability, resulting in extremely important indicators for fatigue detection. An in-depth analysis of the scientific literature allowed us to find the following parameters [5,17,24]: minimum and maximum relative power exerted, mean relative power over time, mean power velocity (MPV), mean and minimum energy.
- *Velocity parameters* [17,24]: useful to understand how easily and quickly the athlete performs the exercise. Minimum and mean velocity, velocity at negative power peak (VNPP), velocity at positive power peak (VPPP) and area under the force-velocity (AFV) curve were analysed.
- *Time parameters* [17,24]: allow us to pinpoint specific events in time during the execution of the exercise (i.e., when the maximum power is exerted or how long the athlete stays in contact with the ground). Therefore, negative power peak time (NPPT), positive power peak time (PPPT) and the total duration of the entire eccentric phase ($t_{ecc}$) were reported in this study.

**Figure 3:**
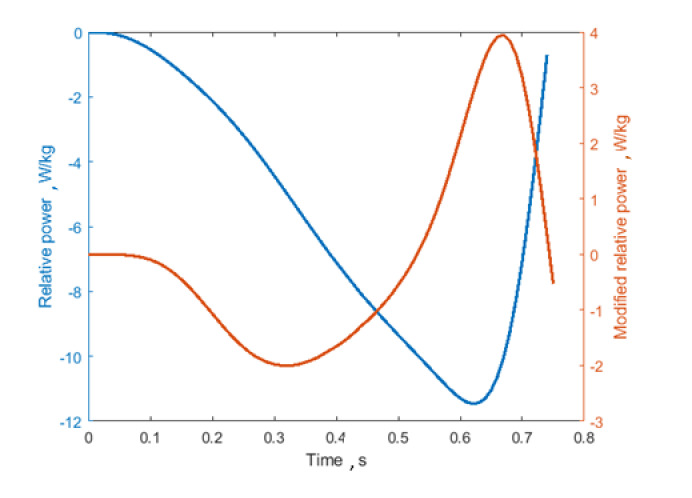
Difference between relative eccentric power (in blue) and modified relative eccentric power (in orange), from which the aforementioned parameters were estimated.

Concerning articular joint kinematic parameters, since the main muscles involved in vertical jumping are hip and knee flexors/extensors, the knee and hip mean flex-extension angular velocities were considered. Similarly to velocity parameters, joint angular velocities could quantify the effort encountered by the athlete during the exercise. In addition, temporal symmetry index (TSI) and duration of the eccentric phase as a percentage of the counter-movement total duration (Eccentric %) were included to monitor how the temporal symmetry of the gesture (eccentric vs. concentric phase) is influenced by fatigue. Although the abovementioned studies estimated the parameters for all the phases of the jump (eccentric + concentric) [13,16], this work focuses on their variation only in the eccentric phase, because it is more susceptible to fatigue and risk injury [25]. Furthermore, as mentioned before, the COM kinematic parameters were estimated starting from the inertial acceleration only (see Table 1) to extract the contribution of the countermovement, neglecting the influence of gravity. This correction led to a different definition of the relative eccentric power curve, which was estimated from the inertial acceleration only. The difference between the commonly adopted power curve and the modified version proposed here is shown in Figure 3. Removing the gravitational component introduces two “bumps”, a negative and a positive one in the curve, with zero crossings in correspondence to the points with either zero velocity or zero acceleration in the eccentric phase (see Figure 2a).

As previously mentioned, two categories of parameters were considered: COM kinematic, which refers to the centre of mass of the athlete’s body, and articular joint kinematic (Table 1).

COM kinematic parameters were estimated by processing on “Matlab” (Mathworks, Natick, MA, USA), the measurement of acceleration of the MIMU placed in correspondence of the fifth lumbar vertebra using formulas reported in Table 1. As concerns the articular joint kinematic parameters, output quaternions of the MIMUs were considered. Once the orientations of each segment were defined, the quaternions associated to the lower limb joints were estimated as the Hamilton product between the distal segment quaternion and the conjugate of the proximal one (1). Finally, joint cardan angles were obtained from the joint quaternion using the relations introduced in [26]. Joint angular velocities were estimated as the temporal derivative of joint cardan angles.

(1) $$q_{joint}=(q_{distal})⨂{\left(q_{proximal}\right)}^{-1}$$

**Figure 4:**
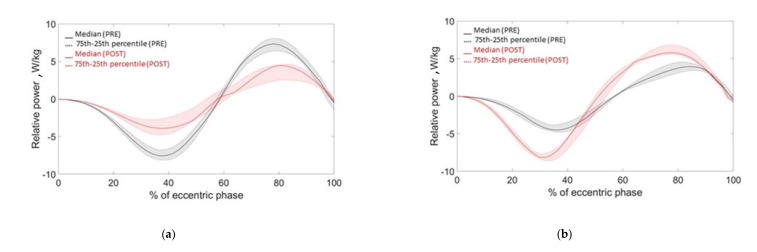
Power traces of a fatigued group (**a**) and Post-Activation Potentiation (PAP) group (**b**) participants.

A preliminary comparison of modified power parameters before and after fatigue induction allowed us to understand if the subject was either in the fatigue or in the PAP group: participants who showed a reduction in the relative power exerted (symptom of a decreased performance) were grouped as fatigued, while those that showed an increase in the power exerted (e.g., improved performance) were grouped as potentiated (Figure 4). Such grouping at a very early stage of the analysis allowed us to study separately the two groups with opposite behaviour. Relative power was selected as the discriminatory parameter because by definition it is calculated on two quantities that should be more influenced by fatigue [27]: relative force and velocity (see Table 1). Indeed, we can notice in Figure 4, which reports the power traces of a representative subject of each group, that the differences between the power curves before and after the YYIR1 are evident, allowing the classification of the two groups. Since one of the objectives of this study is also to quantify the inducted fatigue, a “fatigue approximation index” (FAI) can be proposed by the following definition:

(2) $$FAI\;=\;\frac{p_{post}}{p_{pre}}$$

with:

(3) $$p_{pre}=\;Max\;power_{pre}-Min\;power_{pre}$$

(4) $$p_{post}=\;Max\;power_{post}-Min\;power_{post}$$

where Max power and Min power are the power positive and negative peaks in the eccentric phase of the jump, respectively (Figure 4). In other words, FAI could be interpreted as the ratio between the range of the exerted power (lower-body explosive ability) before and after fatigue induction.

After this initial partition, normalized differences (i.e., $\frac{post\;-\;pre}{pre}$) in parameters before and after fatigue induction were calculated. Once the non-normality distribution of the parameters was verified by using the Kolmogorov–Smirnov test, the one-sample Wilcoxon signed rank test was carried out on the normalized variations to verify their statistical significance. Finally, the Cliff’s Delta (e.g., an effect size test for non-normal distributions) was used to understand the relevance of fatigue influence on performance. The Spearman’s correlation coefficient between FAI and the aforementioned normalized variations of parameters in the fatigued group was calculated to verify if this index could be used as fatigue quantifier. Indeed, the presence of a significant and relevant correlation could give useful information on how the FAI is able to correctly represent the parameter variations and, therefore, if it could be used for fatigue assessment ion behalf of the above-mentioned parameters. In order to find possible correlations between variations of the kinematic parameters and physiological/anthropometric features, Spearman’s correlation coefficient for non-normal distributions was used. The aforementioned tests were carried out with the software “Matlab” (The Mathworks Inc., Natick, MA, USA) assuming a significance threshold α = 0.05.

## 3. Results

Preliminary analysis on power parameters showed that eight out of sixteen athletes presumably underwent in a fatigue condition after the YYIR1, while the remaining participants presumably underwent performance potentiation (PAP). For all the tested parameters in both groups, the Kolmogorov–Smirnov test rejected the null hypothesis that they came from a standard normal distribution for all the sixteen participants. The effects of the YYIR1 test on the two groups (e.g., fatigue group and PAP group) is reported separately in the text below.

### 3.1. Fatigue Group

**Figure 5:**
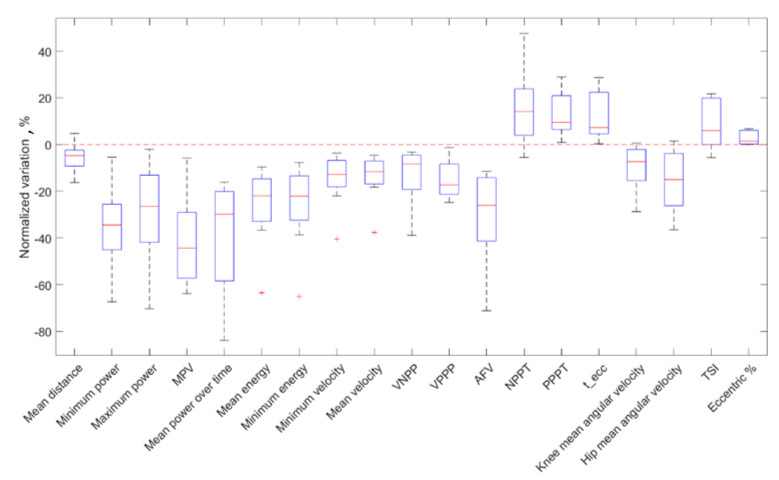
Normalized variations for all the parameters of the fatigued group. MPV = mean power velocity, VNPP = velocity at negative power peak, VPPP = velocity at positive power peak, AFV = area under the force-velocity curve, NPPT = negative power peak time, PPPT = positive power peak time, TSI = temporal symmetry index.

**Figure 6:**
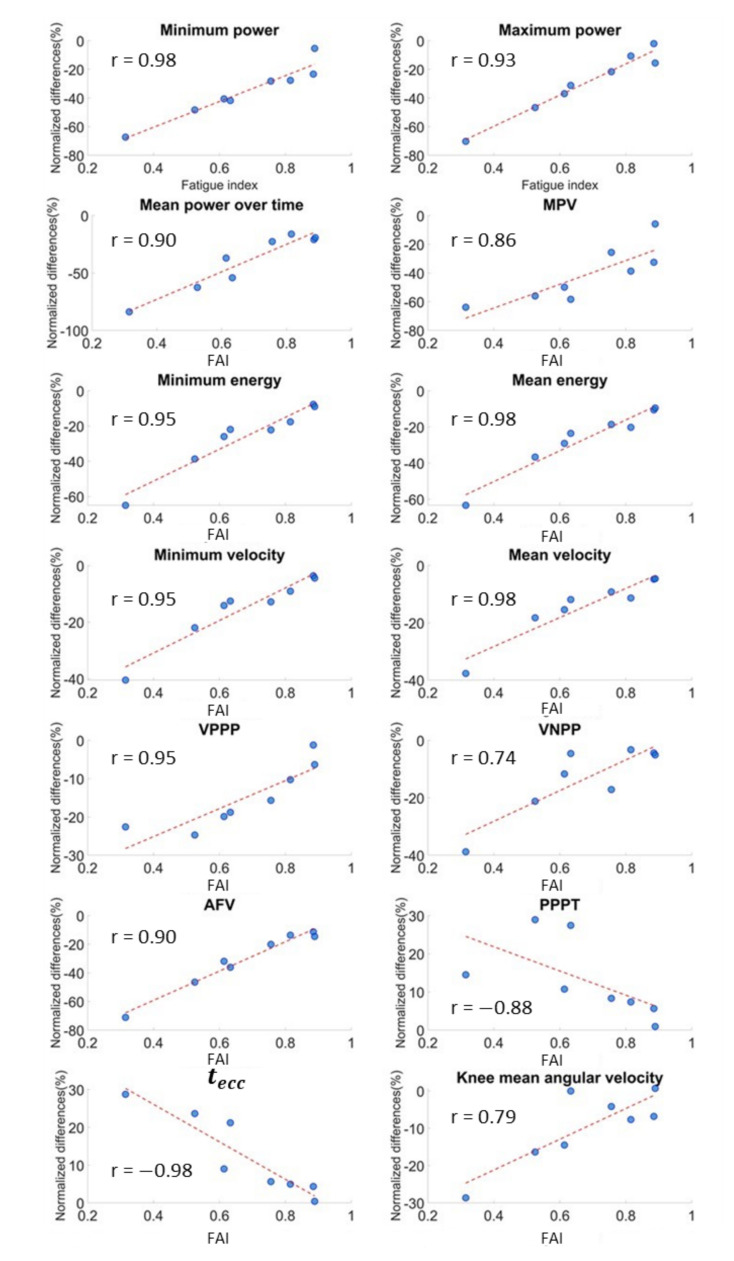
Correlation between fatigue approximation index (FAI) and parameter variations.

Eight out of sixteen athletes showed a reduction in the jump performance. In terms of parameter variations, the execution of a heavy conditioning exercise caused a reduction in counter-movement velocity, exerted power, released energy and articular joint velocities and an increase in execution and contact times (Table 2, Figure 5) as a result. In other words, the athlete was slower and weaker after the YYIR1. All the above parameters had statistically significant variations (*p* < 0.05). According to Cliff’s Delta test, a heavy exercise had generally medium effects (M) on kinematic parameters. Parameter variations are reported in Table 2, together with the FAI Spearman’s correlation coefficient (Figure 6). As stated before, the power parameter variations were wider than in other parameters: a mean absolute normalized variation of 20.5% was detected. Finally, the correlation coefficients resulting from our analysis between anthropometric and kinematic parameters are reported in Table 3. Furthermore, no significant correlations between blood lactate level and kinematic parameters were detected.

### 3.2. PAP Group

**Figure 7:**
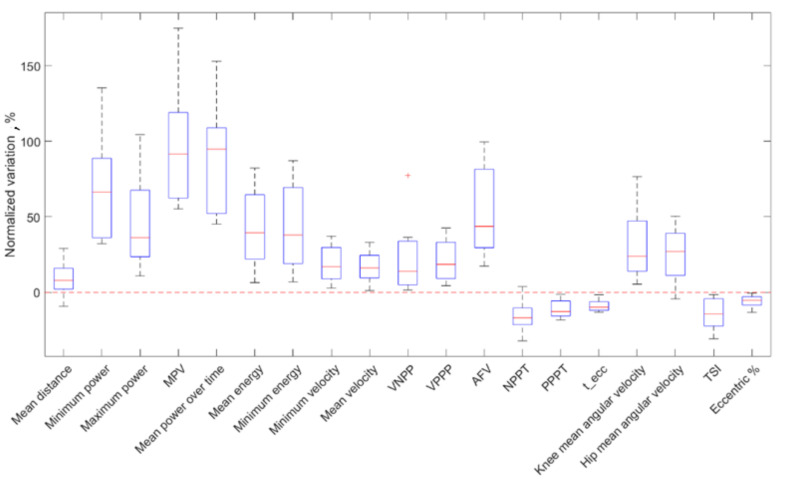
Normalized variations for all the parameters of the PAP group.

The remaining eight participants were characterized by a potentiation. The increase in counter-movement velocity, exerted power, released energy and articular joint velocities and the decrease in execution and contact times were registered (Table 4, Figure 7). Therefore, muscle activation was stronger and faster after the exercise. Here again, all the parameters had statistically significant variations (*p* < 0.05), validating them as indicative factors for performance monitoring, while Cliff’s Delta test revealed generally large effects (L) on kinematic parameters. A mean absolute normalized variation of 34.4% was registered. No significant correlations were observed between anthropometric parameters and kinematic parameters.

## 4. Discussion and Conclusions

The comparison of the values of nineteen kinematic parameters before and after a football-specific fatigue protocol (e.g., YYIR1) confirmed our hypothesis. The Wilcoxon signed rank verified that all the parameters were significantly influenced by the execution of heavy exercise, suggesting that they can be used in a fatigue monitoring solution based on wearable sensors. As mentioned in the Introduction section, two opposites but coherent responses to the fatigue protocol were distinguished. Such differentiation is in accordance with the scientific literature, in which the muscular response is modelled as the contrast between fatigue and potentiation [3]. Therefore, this method successfully allowed us to understand if the athlete was under the positive effect of PAP (e.g., better performance and increased effort tolerance) or under the negative influence of fatigue (e.g., increased risk injury) by only exploiting four wearable sensor (MIMU) and a simple jump test (CMJ). These results are in accordance with those found by Picerno et al. [14], which also validated the Magneto-Inertial technology for vertical jump monitoring. In addition, MIMUs was also used by Chan et al. to detect fatigue conditions by analysing ten kinematic variables of the spine [28] from two inertial units. Similarly to our study, they proposed an index based on the aforementioned ten kinematic variables (Spine Motion Composite Index-SMCI) for spine motion analysis after a fatigue protocol. They found strong correlations between SMCI and objective fatigue measures, showing that MIMUs were a reliable support technology for the detection of variation in spine motion due to muscle fatigue. The same result (i.e., MIMUs as reliable technology for fatigue monitoring in vertical jump) were found in this study as concerns the lower limb, although the correlation with a more objective method (i.e., surface electromyography (sEMG)) is required to validate the FAI as an actual reliable index. Rodacki et al. [11] mainly exploited force plates and electromyography to assess the performance in the vertical jump. They found that peak joint angular velocity, peak joint net moment, and power around the knee were reduced and occurred earlier in comparison with the non-fatigued jumps. In addition, the electromyographic data indicated that the countermovement jumps were performed similarly, so no compensatory movements occurred. sEMG was exploited also by Rahnama et al. [29] to estimate fatigue in athletes after a soccer-specific exercise. It was found that the Root Mean Square (RMS) values of the EMG signals after the fatigue protocol were significantly lower for most of the main lower limb muscles (i.e., rectus femoris, tibialis anterior and biceps femoris). These results confirmed the hypothesis that fatigue induced a reduction in the muscle activation and, therefore, in the exerted force. Nevertheless, considering our specific testing scenario, in which an exhaustive fatigue exercise was proposed, sweat artifacts on EMG data would significantly alter the signal [30], making it impossible to determine if the change in the signal is due to a sweat-related change in electrodes impedance. Among all the above-mentioned studies, Gathercole et al. [17] found variations in the parameters similar to those we found in this work. Similarly to us, they studied the variations in the vertical jump performance in snowboard cross athletes using different technologies, such as a ballistic measurement system, force plates and position transducers. They found that an acute fatigue protocol caused a reduction in the exerted force and an increase in the duration of the jump. We assessed similar results using wearable MIMUs. Indeed, as expected, fatigued athletes showed a reduction in the lower-body explosive ability [24,31], which causes a decreased exerted power, velocity and released energy and increased executions times. In addition, the decrease in joint angular velocities and the resulting increment of the TSI suggest that the athlete is not able to maintain the gesture symmetry due to muscular fatigue. Indeed, the greater duration of the eccentric phase suggests that the athlete requires more time for loading the jump. This phenomenon could be explained by the fact that the fatigue process did not allow us to fully exploit the muscle contraction and, therefore, muscles were not able to take advantage of the elastic force generated by the eccentric movements. Significant correlations were found between muscular mass or weight and the variation of several parameters. Since these correlations involve mainly velocity and power parameters, we can assert that athletes with greater muscular mass lose more of their ability to generate power and maintain higher velocities than those with less lean mass. Unfortunately, no significant correlations between COM kinematic/articular joint kinematic parameters and blood lactate were found, even if all the measured blood lactate levels fall into the normal concentrations range of a football match [32]. The possible reasons of this inconsistency could be that YYIR1 led to a maximal activation of the aerobic system, overshadowing the contribute of the anaerobic system in energy uptake [20]. In this scenario, the blood lactate level might not reflect the real athlete status, becoming an unreliable indicator for fatigue detection and relegating the YYIR1 as a valid test for aerobic analysis [33]. Furthermore, blood lactate level is affected by several external and unpredictable factors (i.e., ambient temperature, hydration status, diet, glycogen content, previous exercise and amount of muscle mass utilized, as well as sampling procedures) [34]), and it may not reflect the real lactate level in the muscles [35].

Conversely, athletes who underwent a potentiation showed the opposite behaviour: increased exerted power, velocity and released energy and decreased executions times. Since higher order muscle fibres are involved in the potentiation, in this case, athletes are faster and the counter-movement gesture is more symmetric (decreased TSI and eccentric duration), while the eccentric phase duration is reduced due to a better elastic force exploitation. In contrast to the first group, in this case, no significant correlations were found between physiological/anatomical parameters and COM kinematic/articular joint kinematic parameters.

Regarding fatigue quantification, the FAI definition was based on the logic presumption that since the exerted power is an indicator of the athlete’s lower-body explosive ability, it could be reasonably considered as the most sensitive parameter to fatigue [27]. Thus, FAI expresses the reduction in this ability due to fatigue induction. In this framework, higher FAI variations are related to a greater fatigued status and vice versa. This phenomenon is confirmed by the correlation coefficients of FAI with regard to the COM kinematic and articular joint kinematic parameters, which were statistically significant for fourteen out of nineteen variables. This suggests the existence of a strong correlation between the “fatigue approximation index” and most of the parameter variations (especially the COM kinematic ones), resulting in a monotonic relation. In this scenario, lower FAI values correspond to higher variations in the parameters and, presumably, to a major fatigue status and vice versa.

This study shows some limitations. Indeed, the participant sample was characterized by a high variability in terms of age and year of agonistics practice, resulting in a heterogeneous study population. In particular, since participants belonged to different training teams, different referees’ preparation could introduce additional variability in the observed data. In addition, the YYIR1 was based on the participants’ subjective perception of fatigue and no physiological parameters were measured to objectively verify the effectivity of the fatiguing exercise. In addition, since the quantification of fatigue is based on external sensors (i.e., Magneto-Inertial Units), the evaluation of fatigue is indirect. The use of sEMG as a wearable alternative could have given useful information on the lower-limb muscles activation and their ability to exert force in association to fatigue. This information could have been compared with the FAI to validate it as an objective index for quantifying fatigue. However, the assessment of fatigue from muscle electrophysiology by means of EMG was impractical, due to the significant sweating of athletes during the proposed test that would result in signal artifacts [30]. Nevertheless, this work also suggests a practical method for performance assessment and fatigue detection/quantification based on four wearable sensors and a simple but reliable CMJ exercise that can also be exploited in uncontrolled environments, allowing outdoor and in-field monitoring. Finally, if we limit our analysis to the COM kinematic parameters, the body inertial sensor network could be optimized from four to two units (i.e., only pelvis and right foot). This reduction, still preserving the capability to identify fatigue-related markers, would make the system more cost effective and accessible for teams of all levels. Future work will be focused on the implementation of the proposed method directly on the pitch (i.e., an uncontrolled environment) during a soccer match to also validate the system for in-field application. In particular, this application will surely give useful information on how fatigue could arise in a real situation (i.e., a 90 min soccer match) and on its possible correlation with the performance and motivation of the referee.

## Figures and Tables

**Table 1:** Main formulas for parameters estimation.

*Physical Quantities*	*MIMU*
Measured quantity	$a_{INERTIAL}=a_{MIMU}-g$
$\mathrm{Velocity}\;(v\left(t\right))$	$v\left(t\right)=\int a_{INERTIAL}*dt$
$\mathrm{Relative}\;\mathrm{power}\;(\frac{P\left(t\right)}{m})$ m=mass	$\frac{P\left(t\right)}{m}=a_{INERTIAL}*v\left(t\right)$
$\mathrm{Mean}\;\mathrm{relative}\;\mathrm{power}\;\mathrm{over}\;\mathrm{time}\;({\left(\frac{P\left(t\right)}{m}\right)}_{mean})$ m=mass	${\left(\frac{P\left(t\right)}{m}\right)}_{mean}=\frac{\frac{P\left(t\right)}{m}}{t_{ecc}}$
$\mathrm{Mean}\;\mathrm{power}\;\mathrm{velocity}\;{\left(v_{p}\right)}_{mean}$	${\left(v_{p}\right)}_{mean\;}=mean(\frac{d}{dt}\frac{P\left(t\right)}{m})$
$\mathrm{Relative}\;\mathrm{energy}\;(\frac{U\left(t\right)}{m})$ m=mass	$\frac{U\left(t\right)}{m}=\;\int \frac{P\left(t\right)}{m}*dt$
$\mathrm{Distance}\;(x\left(t\right))$	$x\left(t\right)=\int \int a_{INERTIAL}*dt^{2}$
$\mathrm{Angular}\;\mathrm{velocities}\;(w_{FE})$	$w_{FE}=\frac{d}{dt}\theta_{FE}$
TSI	$TSI=\left|\frac{t_{conc}-t_{ecc}}{t_{conc}+t_{ecc}}\;\right|$
Eccentric %	${\%}_{ecc}=\left|\frac{t_{ecc}}{t_{conc}+t_{ecc}}\right|$

MIMU = Magneto-Inertial Measurement Unit, TSI = temporal symmetry index.

**Table 2:** Kinematic parameters (fatigued group).

Parameters	PRE	POST	Wilcoxon Signed Rank Test Significance	Normalized Variations (%)	FAI Spearman Coefficient	Cliff’s Delta ES
	$Median\;\left(iqr\right)$	$Median\;\left(iqr\right)$		$Median\;\left(iqr\right)$		
Mean distance (m)	−1.85 (0.54)	−1.76 (0.46)	0.04 *	−4.27 (4.80)	0.12	0.19 (T↓)
$\mathrm{Minimum}\;\mathrm{power}\;(W\cdot kg^{-1})$	−4.92 (5.42)	−3.33 (3.30)	<0.01 **	−34.4 (19.76)	0.98 **	0.38 (**M↓**)
$\mathrm{Maximum}\;\mathrm{power}\;(W\cdot kg^{-1})$	5.42 (5.85)	3.84 (4.56)	<0.01 **	−26.4 (28.8)	0.93 **	−0.34 (**M↓**)
$\mathrm{Mean}\;\mathrm{power}\;\mathrm{over}\;\mathrm{time}\;(W\cdot s^{-1})$	0.26 (0.62)	0.15 (0.34)	<0.01 **	−29.8 (38.2)	0.90 **	−0.38 (**M↓**)
$\mathrm{MPV}\;(W\;\cdot s^{-1})$	−28.3 (39.0)	−15.5 (21.4)	<0.01 **	−44.3 (28.0)	0.86 **	0.47 (**M↓**)
Mean energy ($J\cdot kg^{-1}$)	−3.57 (0.62)	−2.93 (0.74)	<0.01 **	−21.9 (18.3)	0.98 **	0.37 (**M↓**)
Minimum energy ($J\cdot kg^{-1}$)	−7.88 (5.12)	−5.97 (5.00)	<0.01 **	−22.1 (19.1)	0.95 **	0.34 (**M↓**)
$\mathrm{Minimum}\;\mathrm{velocity}\;(m\cdot s^{-1})$	−1.24 (0.18)	−1.08 (0.44)	<0.01 **	−12.7 (11.3)	0.95 **	0.34 (**M↓**)
$\mathrm{Mean}\;\mathrm{velocity}\;(m\cdot s^{-1})$	−0.70 (0.16)	−0.64 (0.22)	<0.01 **	−11.1 (9.84)	0.98 **	0.44 (**M↓**)
$\mathrm{VNPP}\;(m\cdot s^{-1})$	−0.91 (0.38)	−0.82 (0.36)	<0.01 **	−8.37 (14.7)	0.74 *	0.28 (**S↓**)
$\mathrm{VPPP}\;(m\cdot s^{-1})$	−0.82 (0.38)	−0.64 (0.34)	<0.01 **	−17.2 (12.9)	0.95 **	0.31 (**M↓**)
$\mathrm{AFV}\;(N\cdot m\cdot s^{-1}\cdot kg^{-1})$	−15.00 (15.9)	−10.10 (11.4)	<0.01 **	−26.0 (27.2)	0.90 **	0.34 (**M↓**)
NPPT (ms)	20.25 (5.44)	23.25 (3.38)	0.04 *	14.2 (19.98)	−0.19	0.5 (**L↑**)
PPPT (ms)	47.75 (15.8)	57.75 (15.9)	<0.01 **	9.53 (14.4)	−0.88 **	0.25 (**S↑**)
$t_{ecc}$ (ms)	55.00 (15.8)	60.00 (16.1)	<0.01 **	7.32 (17.8)	−0.98 **	0.28 (**S↑**)
Knee mean angular velocity (deg/s)	113.9 (31.8)	107.8 (29.2)	0.02 *	−7.23 (9.20)	0.79 *	−0.39 (**M↓**)
Hip mean angular velocity (deg/s)	114.7 (56.2)	101.9 (28.2)	0.02 *	−16.2 (13.2)	0.21	−0.55 (**L↓**)
TSI	0.419 (0.10)	0.488 (0.18)	0.02 *	6.04 (18.5)	−0.57	0.43 (**M↑**)
Eccentric %	0.711 (0.04)	0.744 (0.08)	0.02 *	1.51 (5.04)	−0.43	0.43 (**M↑**)

* = *p* < 0.05, ** = *p* < 0.01, *iqr* = interquartile range, ES = effect size, T = trivial, S = small, M = moderate, L = large. FAI = fatigue approximation index, MPV = mean power velocity, VNPP = velocity at negative power peak, VPPP = velocity at positive power peak, AFV = area under the force-velocity curve, NPPT = negative power peak time, PPPT = positive power peak time, TSI = temporal symmetry index.

**Table 3:** Correlations between centre of mass (COM) kinematic parameters and anthropometric ones.

Parameters	Weight	Muscular Mass
Maximum power	-	0.83 *
Minimum energy	0.79 *	0.88 **
Minimum velocity	0.79 *	0.88 **
VPPP	-	0.82 *
VNPP	0.86 *	0.83 **

* = *p* < 0.05, ** = *p* < 0.01. VNPP = velocity at negative power peak, VPPP = velocity at positive power peak.

**Table 4:** Kinematic parameters (PAP group).

Parameters	PRE	POST	Wilcoxon Signed Rank Test Significance	Normalized Variations (%)	Cliff’s Delta ES
	$Median\;\left(iqr\right)$	$Median\;\left(iqr\right)$		$Median\;\left(iqr\right)$	
Mean distance (m)	−1.27 (0.30)	−1.31 (0.32)	0.08	7.98 (13.8)	−0.19 (**T↑**)
Minimum power (W)	−2.77 (1.38)	−4.34 (1.96)	<0.01 **	66.4 (52.6)	−0.78 (**L↑**))
Maximum power (W)	3.47 (1.26)	4.66 (1.74)	<0.01 **	36.3 (44.2)	0.72 (**L↑**)
$\mathrm{Mean}\;\mathrm{power}\;\mathrm{over}\;\mathrm{time}\;(W\cdot dt^{-1})$	0.16 (0.08)	0.34 (0.20)	<0.01 **	94.8 (57.0)	0.72 (**L↑**)
$\mathrm{MPV}\;(W\cdot dt^{-1})$	−14.37 (6.80)	−28.83 (14.0)	<0.01 **	91.6 (56.6)	−0.81 (**L↑**)
Mean energy (J)	−2.02 (0.66)	−2.49 (0.378)	<0.01 **	39.4 (42.6)	−0.62 (**L↑**)
Minimum energy (J)	−4.36 (1.62)	−5.43 (1.76)	<0.01 **	37.8 (50.4)	−0.59 (**L↑**)
$\mathrm{Minimum}\;\mathrm{velocity}\;(m\cdot s^{-1})$	−0.92 (0.18)	−1.03 (0.16)	<0.01 **	16.9 (20.8)	−0.59 (**L↑**)
$\mathrm{Mean}\;\mathrm{velocity}\;(m\cdot s^{-1})$	−0.54 (0.08)	−0.58 (0.10)	<0.01 **	16.2 (14.9)	−0.69 (**L↑**)
$\mathrm{VNPP}\;(m\cdot s^{-1})$	−0.71 (0.16)	−0.78 (0.12)	<0.01 **	13.9 (28.8)	−0.58 (**L↑**)
$\mathrm{VPPP}\;(m\cdot s^{-1})$	−0.60 (0.14)	−0.67 (0.16)	<0.01 **	18.5 (42.0)	−0.63 (**L↑**)
$\mathrm{AFV}\;(N\cdot m\cdot s^{-1})$	−9.55 (3.06)	−13.13 (4.64)	<0.01 **	43.6 (52.2)	−0.84 (**L↑**)
NPPT (ms)	20.38 (3.76)	17.38 (4.50)	0.02 *	−17.0 (11.1)	−0.56 (**L↓**)
PPPT (ms)	44.50 (6.76)	38.88 (11.0)	<0.01 **	−12.9 (9.96)	−0.40 (**M↓**)
$t_{ecc}$ (ms)	50.88 (0.08)	45.25 (10.0)	<0.01 **	−9.92 (5.66)	−0.34 (**M↓**)
Knee mean angular velocity (°/sec)	108.5 (20.5)	124.6 (29.6)	<0.01 **	27.1 (33.4)	0.81 (**L↑**)
Hip mean angular velocity (°/sec)	108.7 (22.8)	131.8 (38.4)	<0.01 **	27.0 (27.2)	0.68 (**L↑**)
TSI	0.486 (0.08)	0.407 (0.12)	<0.01 **	−14.5 (15.6)	−0.50 (**L↓**)
Eccentric %	0.743 (0.04)	0.691 (0.80)	<0.01 **	−5.48 (5.54)	−0.56 (**L↑**)

* = *p* < 0.05, ** = *p* <0.01, *iqr* = interquartile range, ES = effect size, T = trivial, S = small, M = moderate, L = large. PAP = post-activation potentiation, FAI = fatigue approximation index, MPV = mean power velocity, VNPP = velocity at negative power peak, VPPP = velocity at positive power peak, AFV = area under the force-velocity curve, NPPT = negative power peak time, PPPT = positive power peak time, TSI = temporal symmetry index.

## Data Availability

The data presented in this study are available on request from the corresponding author.
